# Supplementary material for: Range Dynamics of Striped Field Mouse (Apodemus agrarius) in Northern Eurasia under Global Climate Change Based on Ensemble Species Distribution Models
Source: Biology (Basel). 2023 Jul 22;12(7):1034. doi: 10.3390/biology12071034 (PMC10376031; doi:10.3390/biology12071034)
Supplement: Supplementary file 1 [file biology-12-01034-s001.zip › biology-2449608-supplementary.pdf]

Supplementary materials

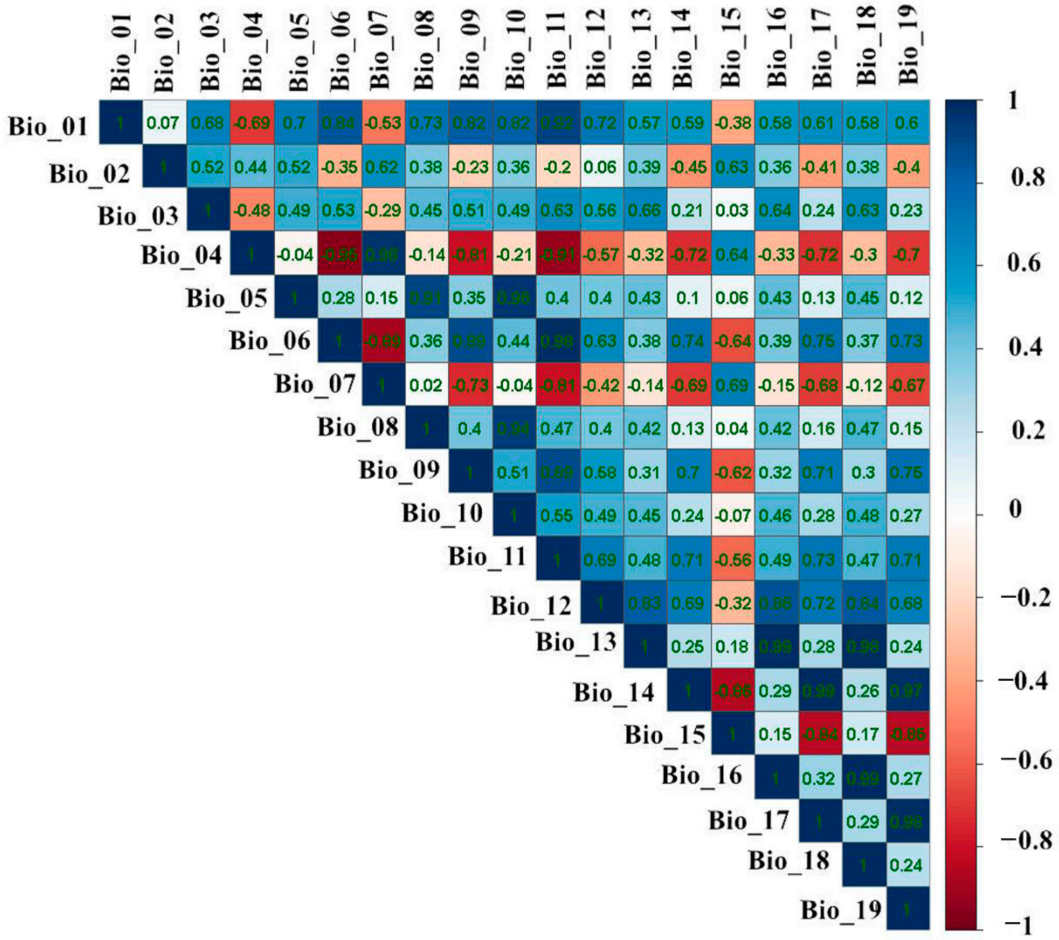

Figure S1: Spearman's correlation matrix heatmap of bioclimatic variables. The variables include: Bio_01 (annual mean temperature), Bio_02 (mean diurnal range (mean of monthly (max temp–min temp))), Bio_03 (isothermality Bio_02/Bio_07) (× 100), Bio_4 (temperature seasonality (standard deviation × 100)), Bio_05 (max temperature of warmest month), Bio_06 (min temperature of coldest month), Bio_07 (temperature annual range (Bio_05–Bio_06)), Bio_08 (mean temperature of wettest quarter), Bio_09 (mean temperature of driest quarter), Bio_10 (mean temperature of warmest quarter), Bio_11 (mean temperature of coldest quarter), Bio_12 (annual precipitation), Bio_13 (precipitation of wettest month), Bio_14 (precipitation of driest month), Bio_15 (precipitation seasonality (coefficient of variation)), Bio_16 (precipitation of wettest quarter), Bio_17 (precipitation of driest quarter), Bio_18 (precipitation of warmest quarter), and Bio_19 (precipitation of coldest quarter).

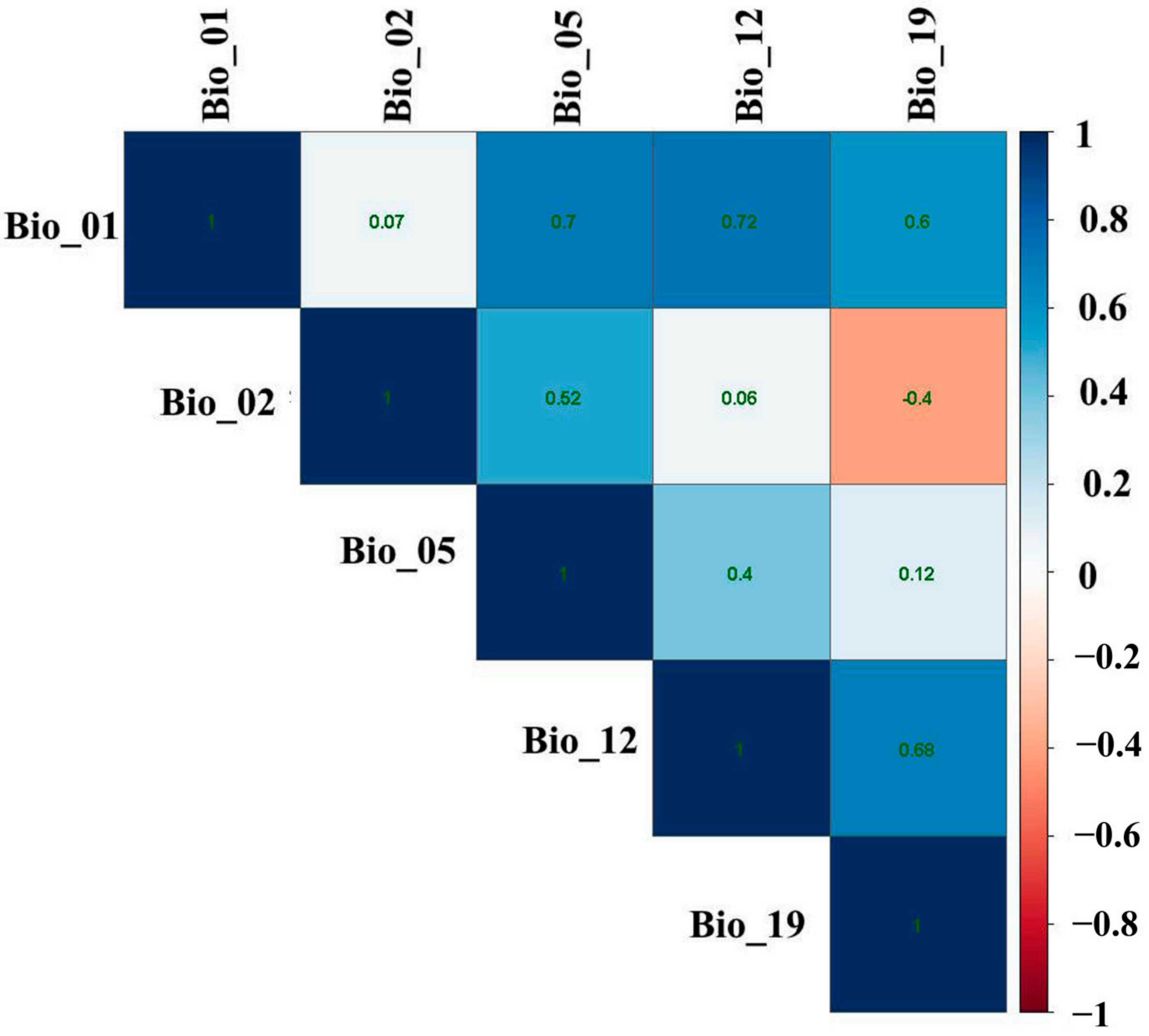

Figure S2. Spearman's correlation matrix heatmap of predictor variables. The variables include: Bio_01 (mean annual temperature), Bio_02 (mean diurnal range of temperature). Bio_05 (maximum temperature of warmest month), Bio_12 (annual precipitation), and Bio_19 (precipitation of coldest quarter).

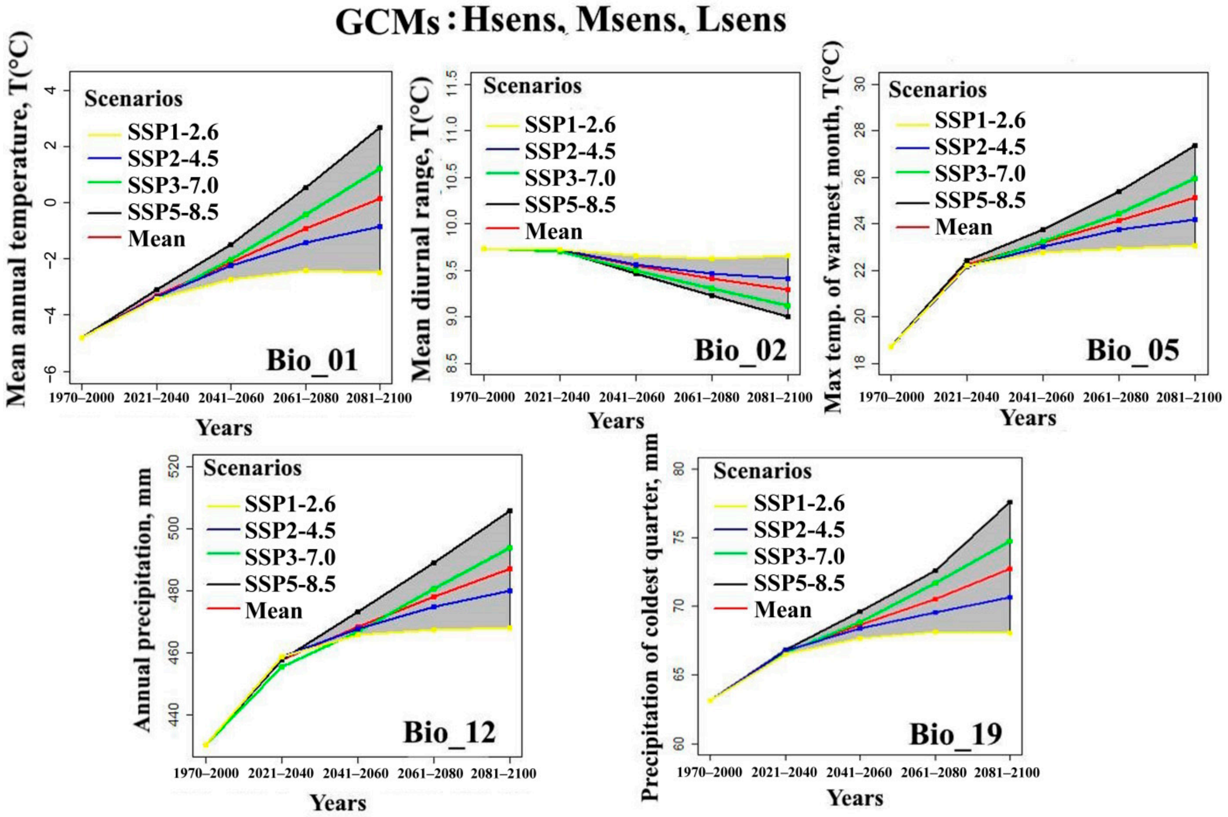

Figure S3. Predicted means of variables averaged for all GCMs under four scenarios of climate change in 2021-2100.

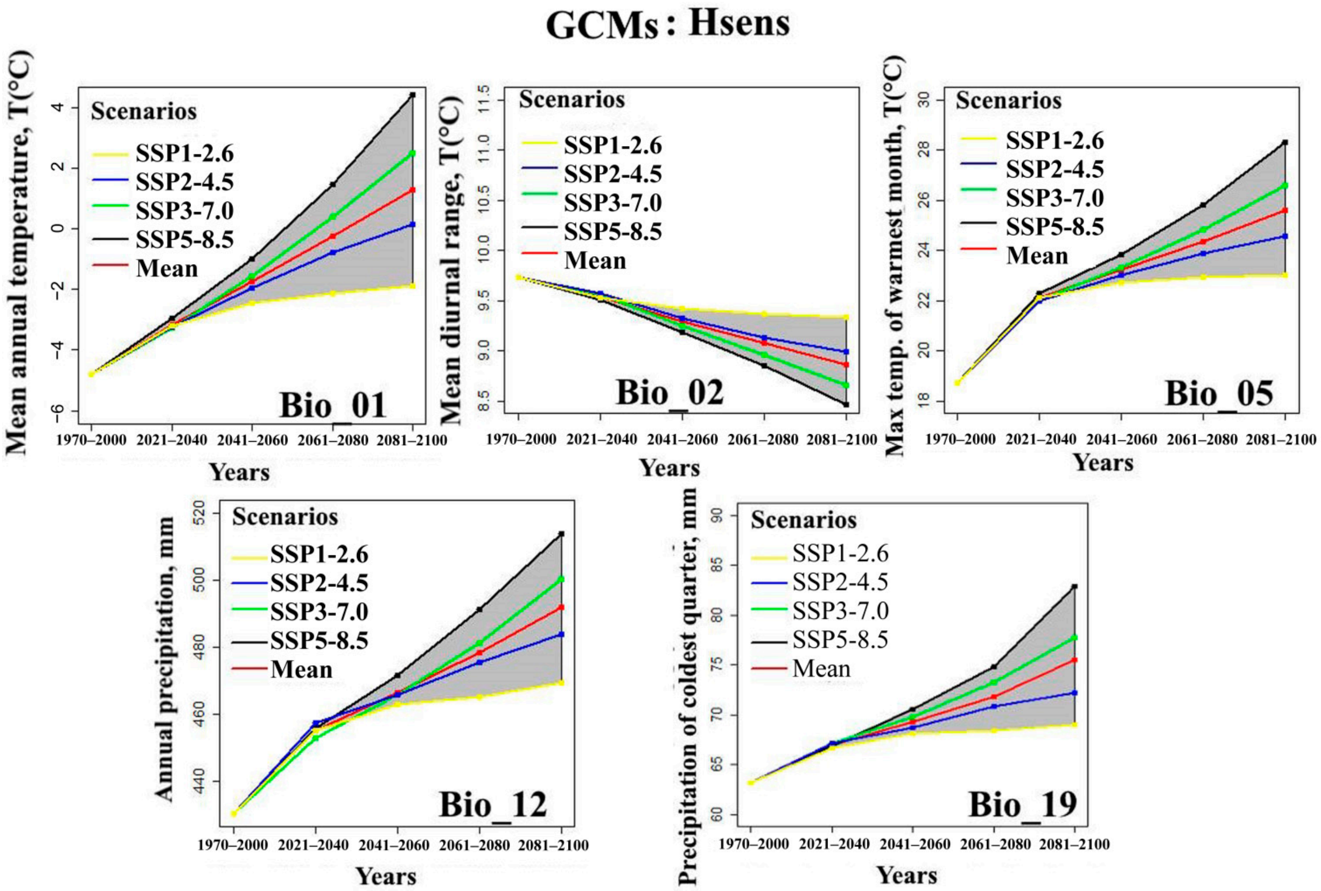

Figure S4. Assessment of the predictor variable means for high-sensitivity (Hsens) GCMs under four climate change scenarios in 2021-2100.

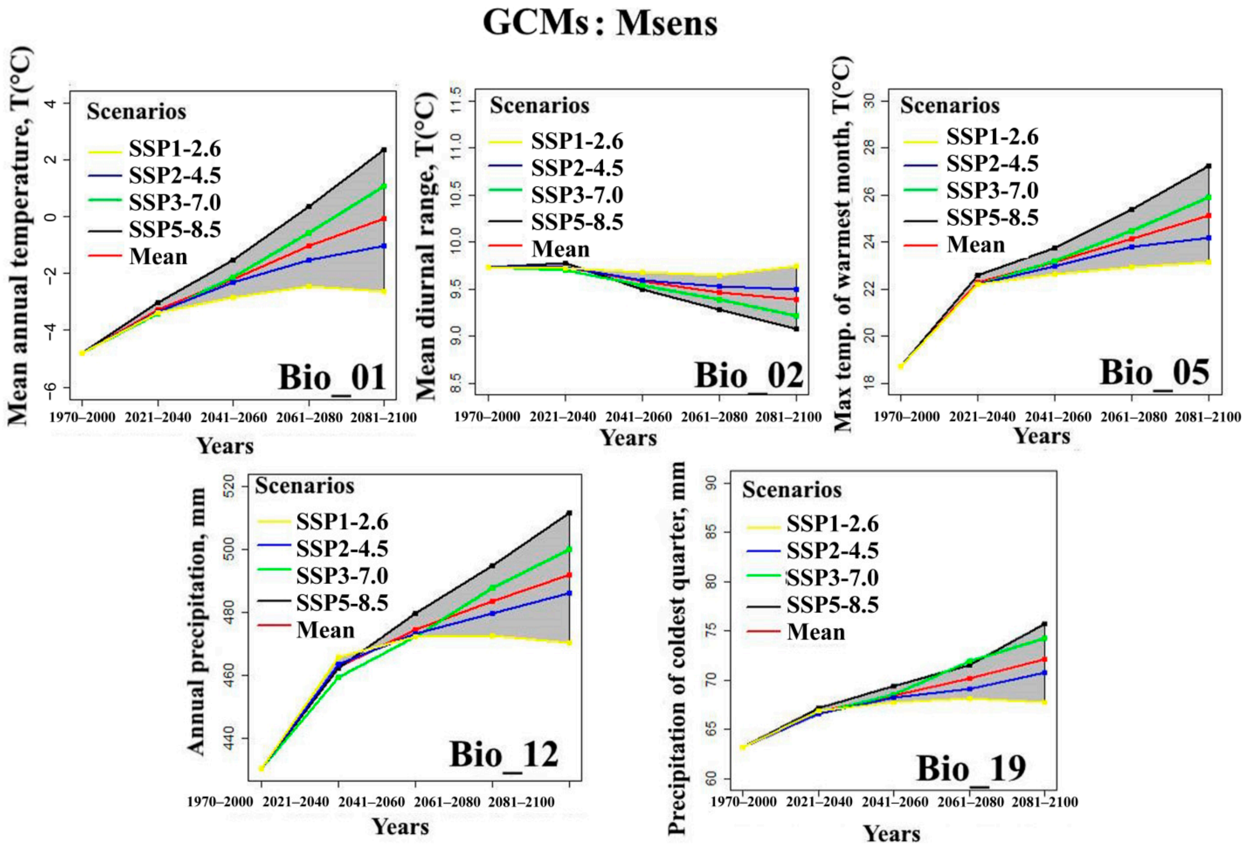

Figure S5. Assessment of the predictor variable means for medium-sensitivity (Msens) GCMs under four climate change scenarios in 2021-2100.

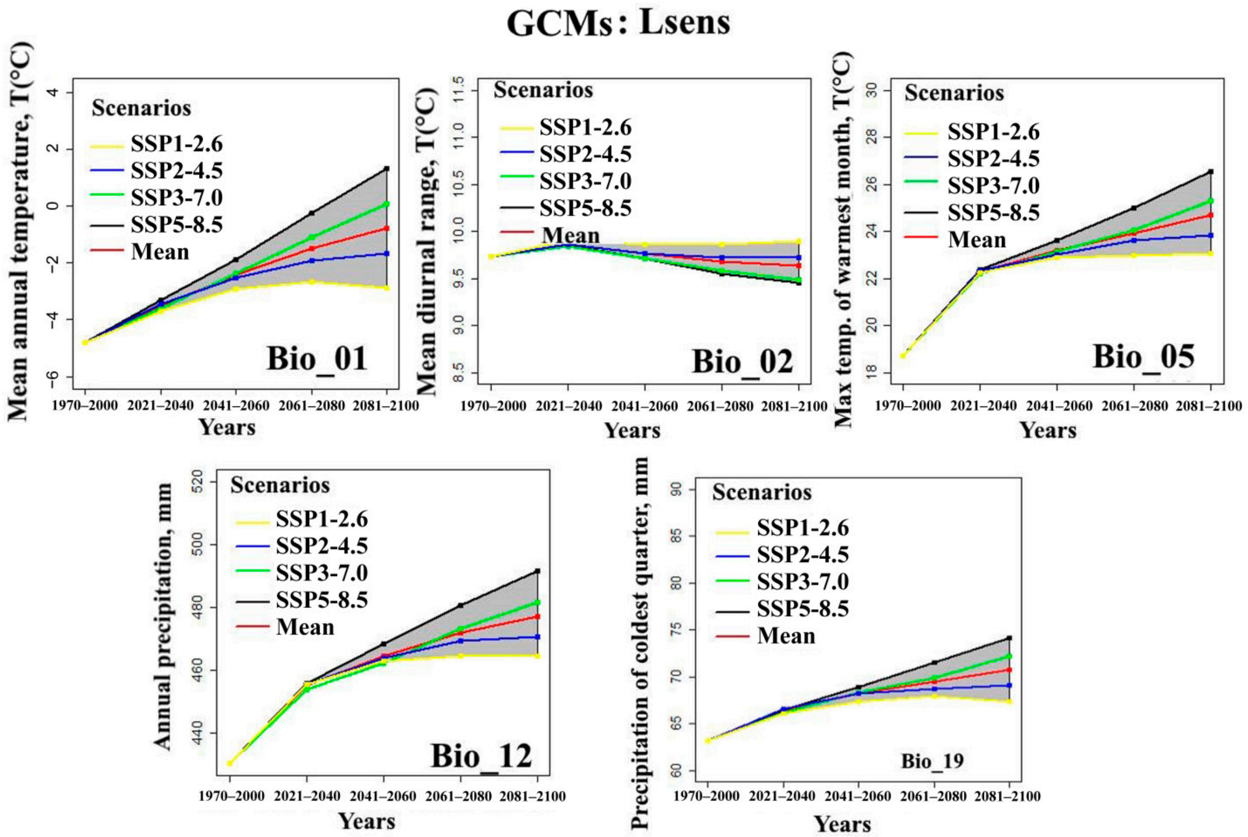

Figure S6. Assessment of the predictor variable means for low-sensitivity (Lsens) GCMs under four climate change scenarios in 2021-2100.

Below is a description of the basic methods and algorithms that were used to determine the optimal parameters of iSDMs (Figures S7–S12).

Determination of the GLMs parameters. For this model, we determined the parameters by analyzing various alternative models using linear, quadratic, or polynomial functions and a stepwise procedure for including variables in the model. The most important parameters defined by *Biomod2_tuning* are: type, interaction.level, myFormula, where type (model type) takes a value from the set (simple (=linear), quadratic, polynomial); interaction.level (interaction level of between considered variables); myFormula (concrete GLM function) is proposed by the *Biomod2_tuning* function to build the optimal model. The rest of the parameters are set by default.

Determination of the GAMs parameters. We defined the GAMs parameters by using the *mgcv* R-package. The R-package automatically fits the response curves "as close as possible" to the original data, taking into account the allowed level of smoothing by internal cross-checking. All parameters are automatically determined by *mgcv*, but one parameter, selection, must be defined. This parameter can take two values - True (1) or False (0). If the parameter takes True (1), the package can add an extra "penalty" to each term, so that it can be penalized to zero. This means that when evaluating the smoothing parameter, which is part of the smoothing fit, some models term may be removed. If the parameter takes the value False (0), the additional "penalty" has no effect. Thus, the degree of terms smoothing is controlled to avoid overtraining the model. However, after performing some computational experiments, we found that *mgcv* does not define the two optimal parameters for k and interaction.level by default. The parameter k is the dimension of the basis used to represent the smooth term, and the parameter interaction.level indicates the degree of interaction of the predictor variables. For this reason, we additionally determined the values of these parameters.

Determination of the GBMs parameters. The *Biomod2_tuning* function with the R-package *gbm* defines the following important parameters: n.trees, interaction.depth, and shrinkage. The parameter n.trees specifies the maximum number of trees to be defined. This parameter is equivalent to the number of iterations required to build consecutive trees. The interaction.depth parameter specifies the number of internal nodes in each tree, which controls the complexity of the tree ensemble. This parameter reflects the depth of interaction between variables in trees. The shrinkage parameter is known as the rate at which the model is trained when the boosting is implemented. This parameter controls the weight given to the consecutive trees. It usually takes values from 0.001 to 0.1, but more trees are usually needed at a lower training rate. The other parameters are defined by default.

Determination of the FDA models parameters. The R-package *mda* is used to determine the FDA models parameters, which does not require many arguments, except for specifying MARS as the fitting method and two additional parameters degree and nprune. The degree parameter is the maximum level of interaction between the variables; if it is 1, then there are no interactions between the variables. The parameter nprune is the maximum number of terms in the reduced model, i.e. which indicates the number of piecewise linear (hinge) functions needed to build the model. If its value is NULL, it means that all the terms created by the forward pass remain in the model. This parameter is used to set an upper limit on the model size and reduce the search time.

Determination of the RF models parameters. The *Biomod2_tuning* function in the *randomforest* R-package determines the following parameters: ntree, mtry, nodesize, maxnodes. The parameter ntree defines the number of model trees. This number should not be too small to ensure that each record will be predicted at least several times. The mtry parameter determines the number of variables randomly chosen as candidates for each splitting. The nodesize parameter specifies the minimum number of terminal nodes. The maxnodes parameter is the maximum number of nodes that trees can have in the forest. If this parameter is not specified, the trees are created to the maximum possible size (taking into account nodesize restrictions).

Determination of the ANN models parameters. The *Biomod2_tuning* function defines the parameters of the neural network using the R-package *nnet*. This package is the most widely used, and easiest to use because it contains only one layer of hidden nodes. Important parameters in nnet are the number of units in a hidden layer (size) and the "weight decay" coefficient, which controls the optimization process and avoids overtraining.

Determination of the MaxEnt models parameters. We used three threshold-independent metrics to determine the optimal MaxEnt parameters: AUC_{Test}, AUC_{Diff}, and Akaike-adjusted "information criterion" (AICc) using the Biomod2_tuning. AUC_{Test} is a metric that measures the discriminative ability of the iSDM using the testing samples (records). AUC_{Diff} is the difference between the AUCs calculated from the AUC_{Train} (training) and AUC_{Test} (testing) samples. This metric (AUC_{Diff}) is a measure of model overfitting. High AUC_{Diff} values characterize a high degree of model overfitting [1]. AICc, the Akaike information criterion, adjusted for small sample size, reflects the goodness-of-fit and complexity [4]. The iSDM with the lowest AICc value is assumed to be the most appropriate model among the set of possible models. The main parameters of MaxEnt evaluated by *Biomod2_tuning* are: regularization multiplier (RM) and classes of predictor functions: linear (L), quadratic (Q), their product/interaction (P), threshold (T) and hinge (H). The RM parameter controls how closely-fitted the output distribution. The default value (RM = 1) can result in a strong localized model distribution that more closely matches the records , but can also lead to model overfitting. The *Biomod2_tuning* function evaluates the above mentioned parameters using the ENMeval R-package [3]. However, the speed of parameter estimation using *Biomod2_tuning* is higher, because it uses multi-threaded calculation mode. The *Biomod2_tuning* function creates a series of MaxEnt models using different RM-values and feature classes, compares them using the AICc criterion and suggests the most appropriate model. This function commonly selects a model that is less complex than the default model accepted by MaxEnt, with an acceptable AUC_{Diff} metric value [4,5,6,7].

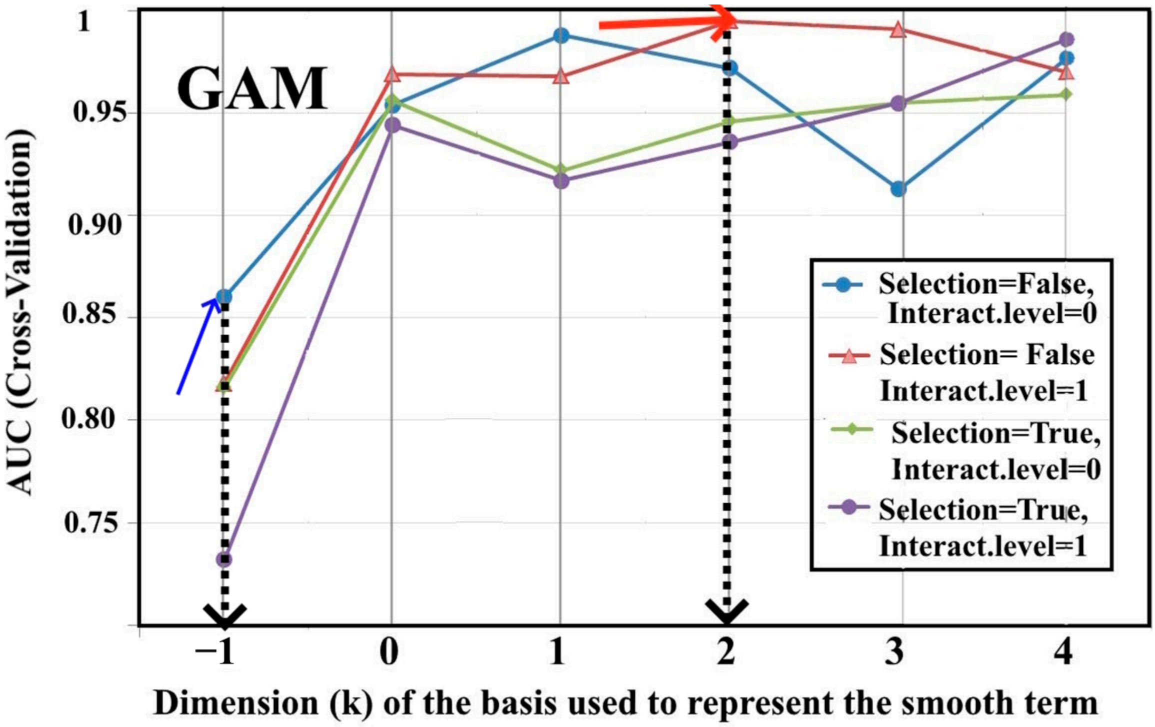

Figure S7. Dependence of the AUC values on GAM parameters (k, interaction.selection). The optimal and default GAM parameters are represented by the red and blue arrows, respectively.

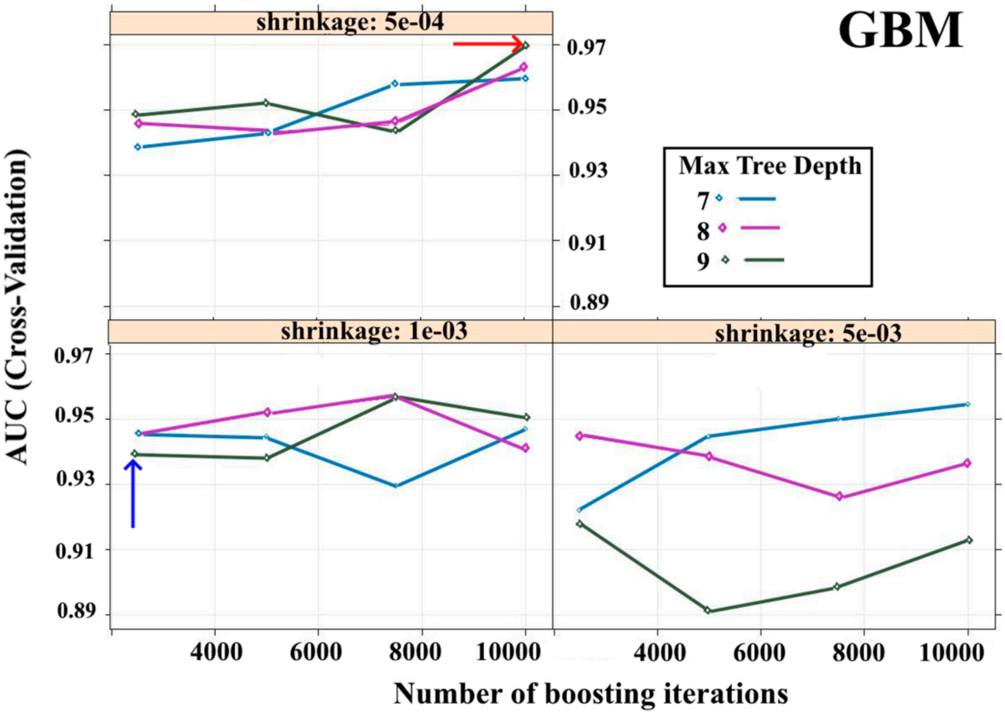

Figure S8. Dependence of the AUC values on GBM parameters (n.trees, interaction.depth, shrinkage). The optimal and default GBM parameters are represented by the red and blue arrows, respectively.

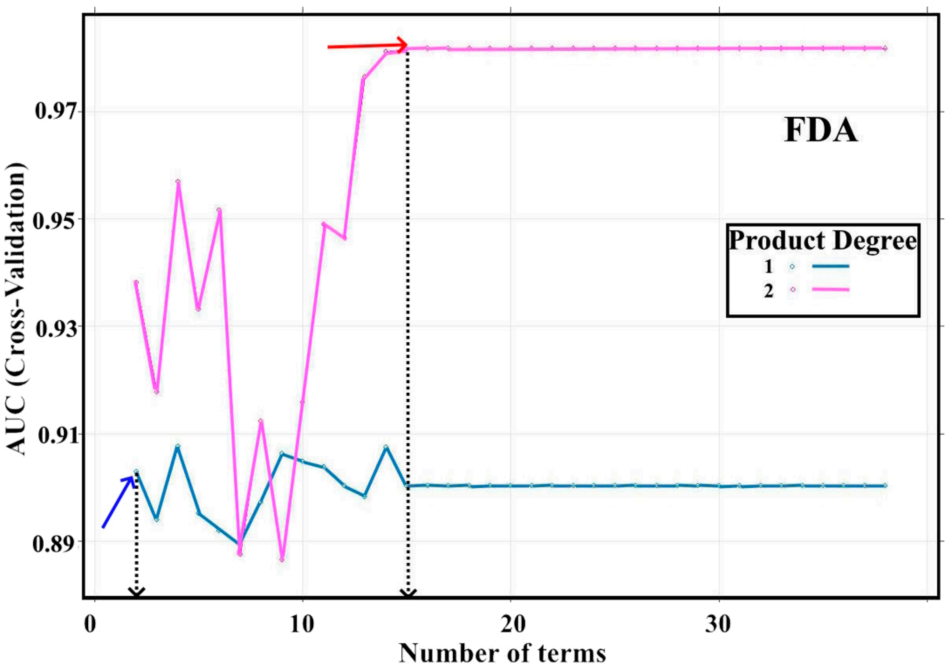

Figure S9. Dependence of the AUC values on FDA model parameters (degree, nprune). The optimal and default FDA model parameters are represented by the red and blue arrows, respectively.

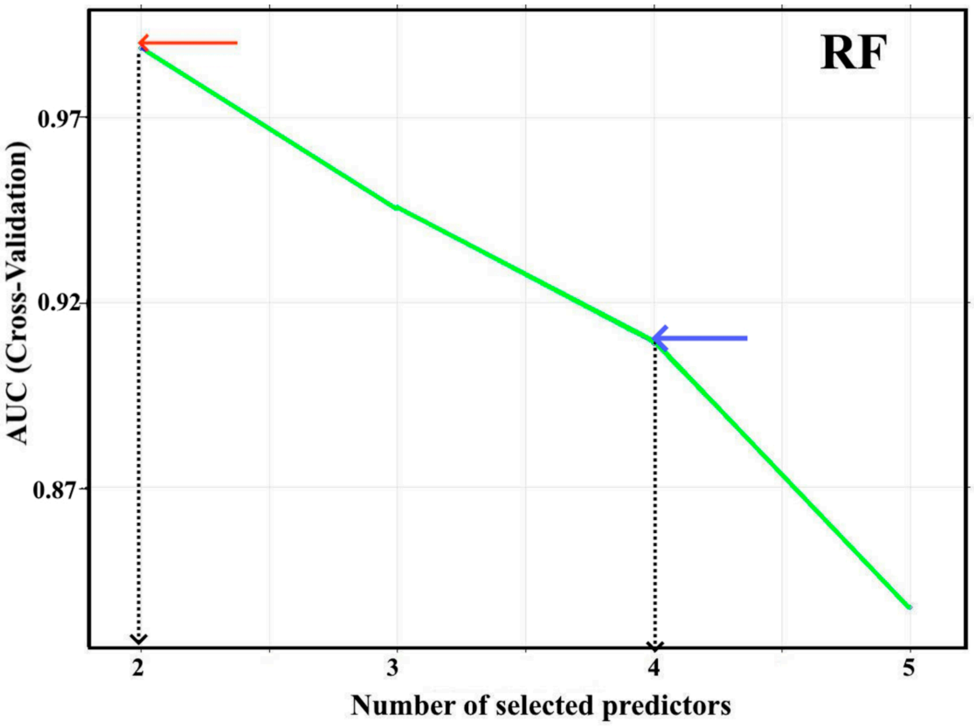

Figure S10. Dependence of the AUC values on RF model parameter (mtry). The optimal and default RF model parameters are represented by the red and blue arrows, respectively.

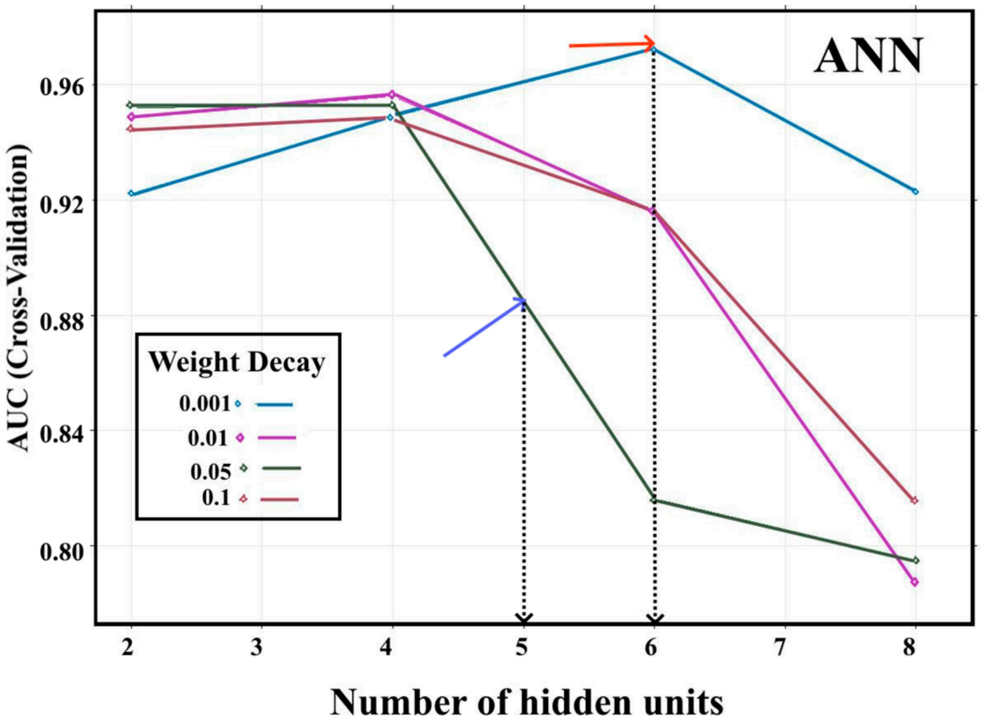

Figure S11. : Dependence of the AUC values on ANN model parameters (size, weight decay). The optimal and default ANN model parameters are represented by the red and blue arrows, respectively.

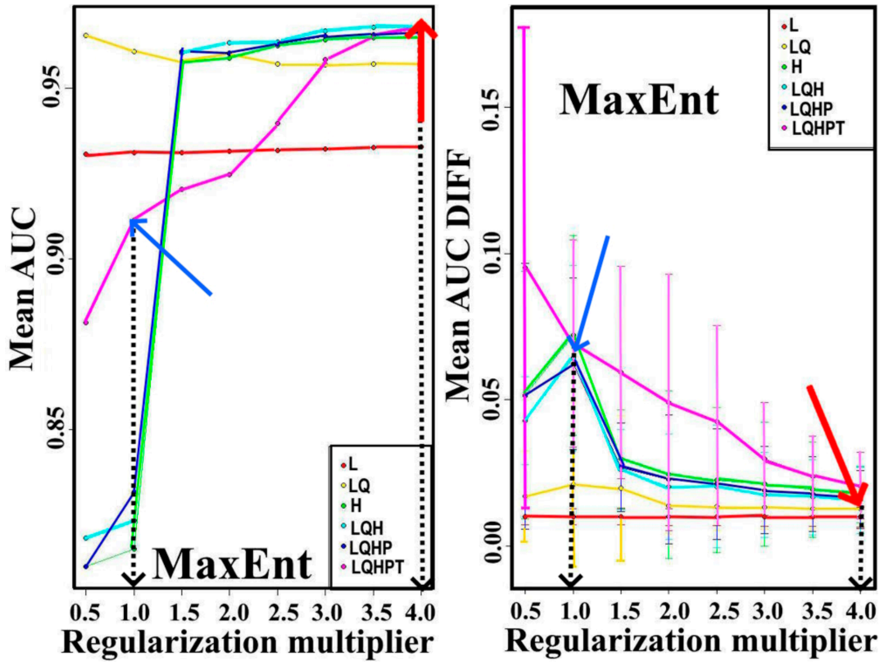

Figure S12. Dependences of average AUC values of MaxEnt models for a number of combinations of feature classes (L = linear, Q = quadratic, P = product, T = threshold, H = hinged) and regularization parameters (RPs). The optimal and default MaxEnt model parameters (combinations of feature classes and regularization parameters) are indicated by the red and blue arrows, respectively.

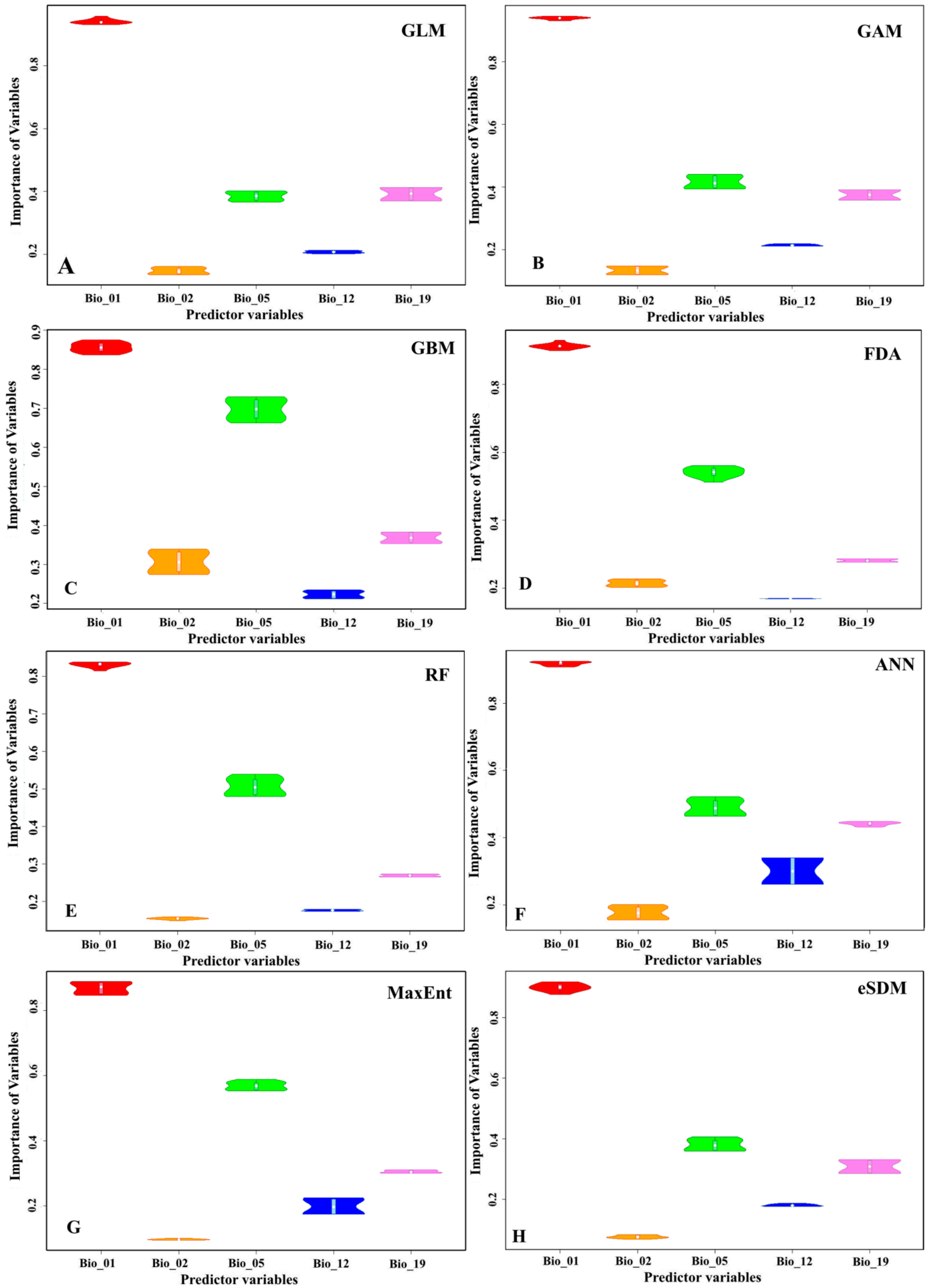

Figure S13. Violin plots of the variables importance in the created individual GLM (A), GAM (B), GBM (C), FDA (D), RF (E), ANN (F), MaxEnt (G) and eSDM (H) under current climate condition.

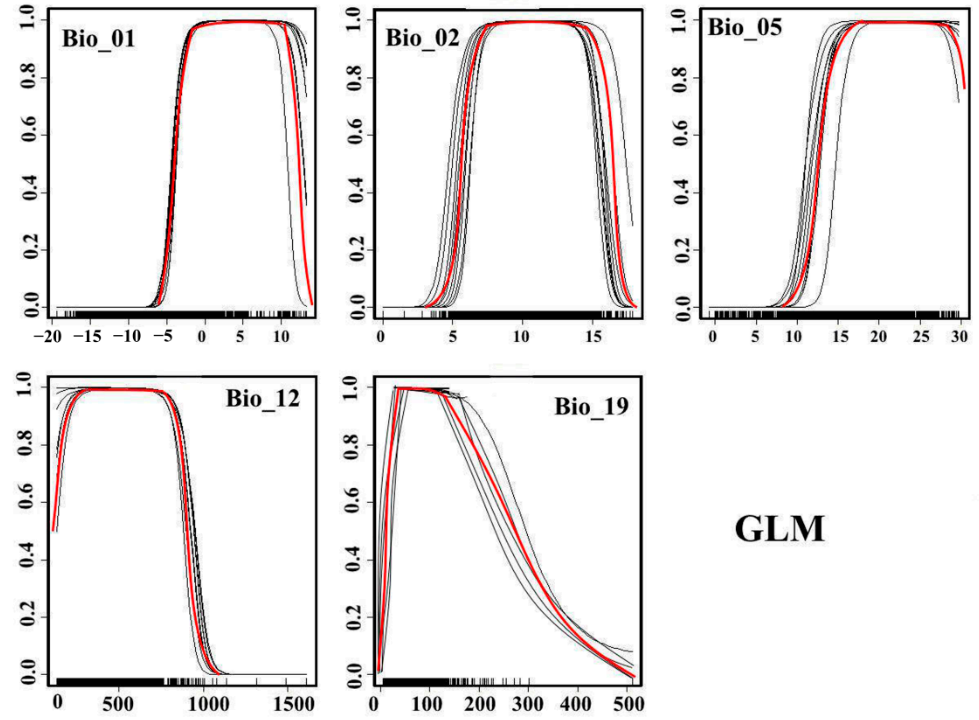

Figure S14. Plot of the response curves of five variables in GLMs for *Apodemus agrarius*.

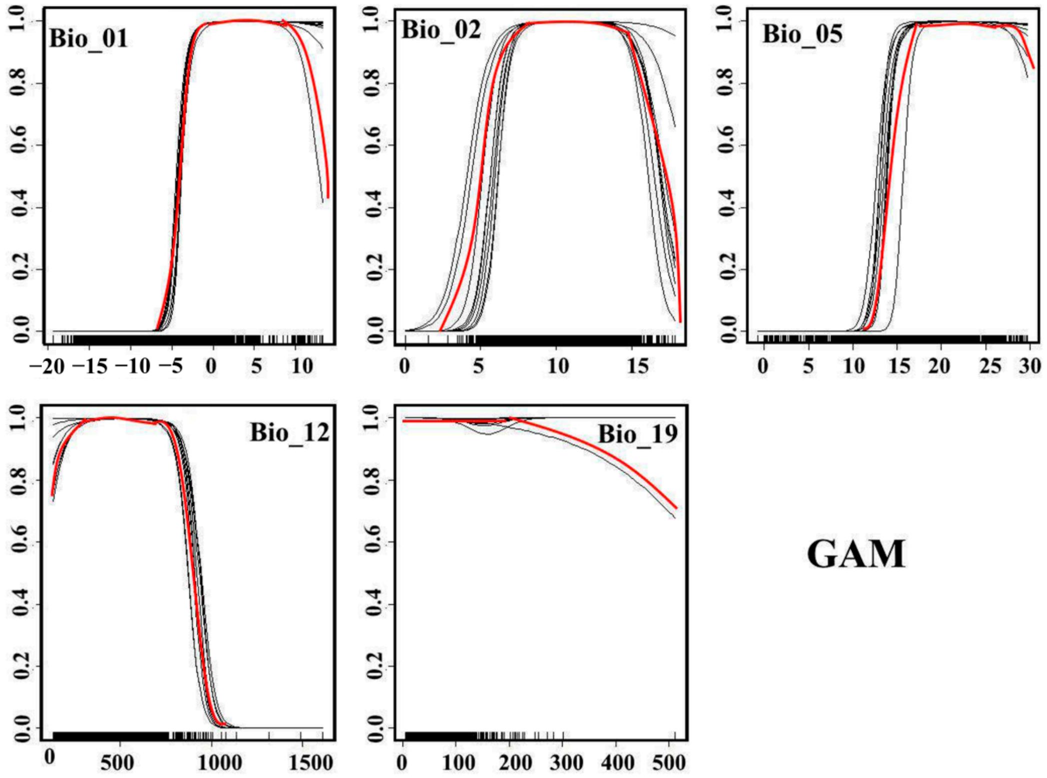

Figure S15. Plot of the response curves of five variables in GAMs for *Apodemus agrarius*.

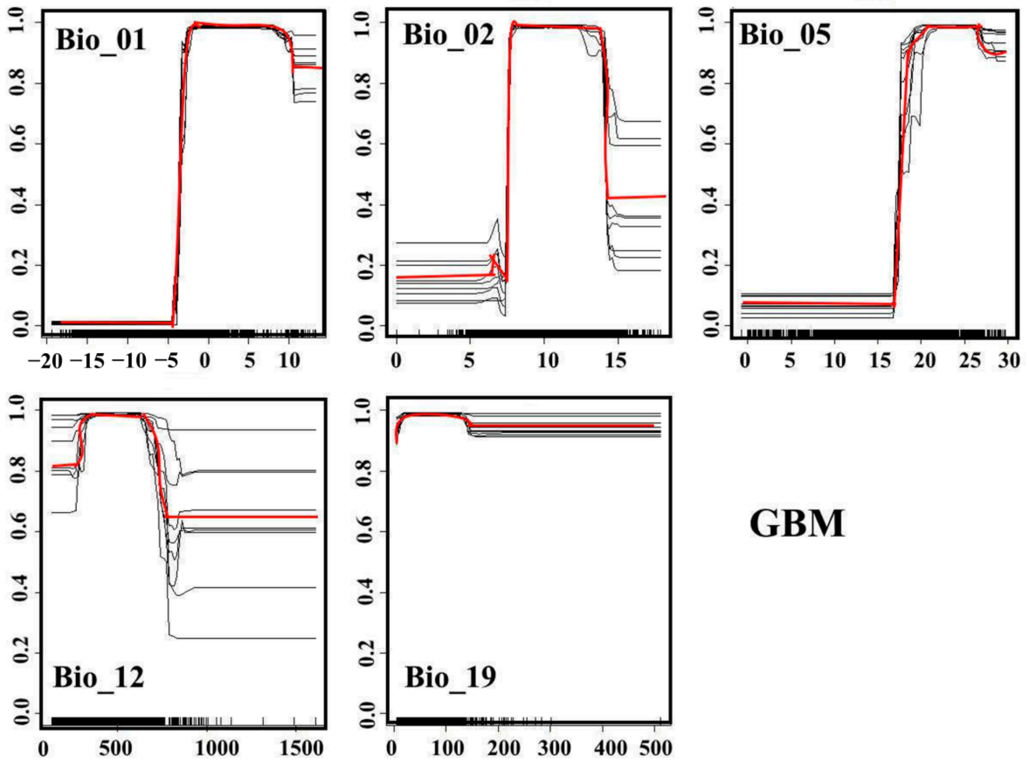

Figure S16. Plot of the response curves of five variables in GBMs for *Apodemus agrarius*.

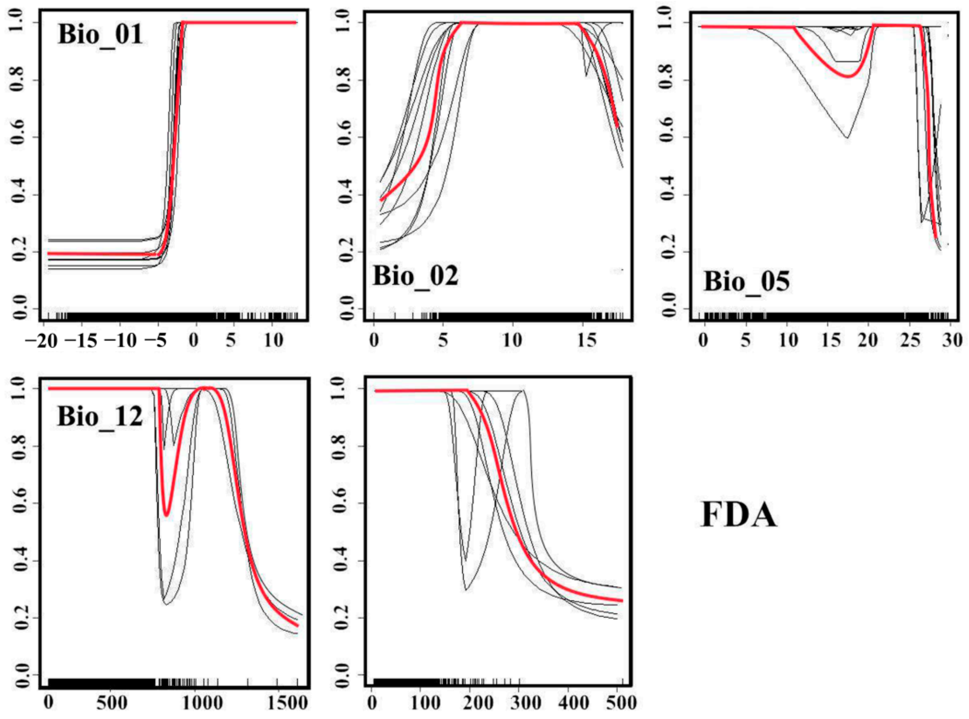

Figure S17. Plot of the response curves of five variables in FDA models for *Apodemus agrarius*.

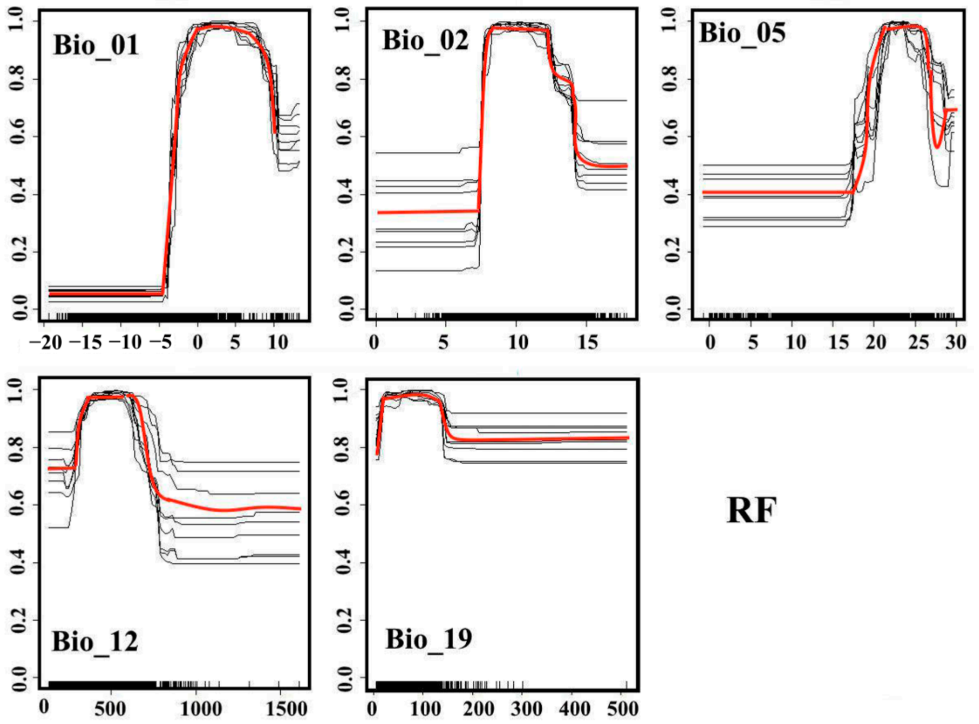

Figure S18. Plot of the response curves of five variables in RF models for *Apodemus agrarius*.

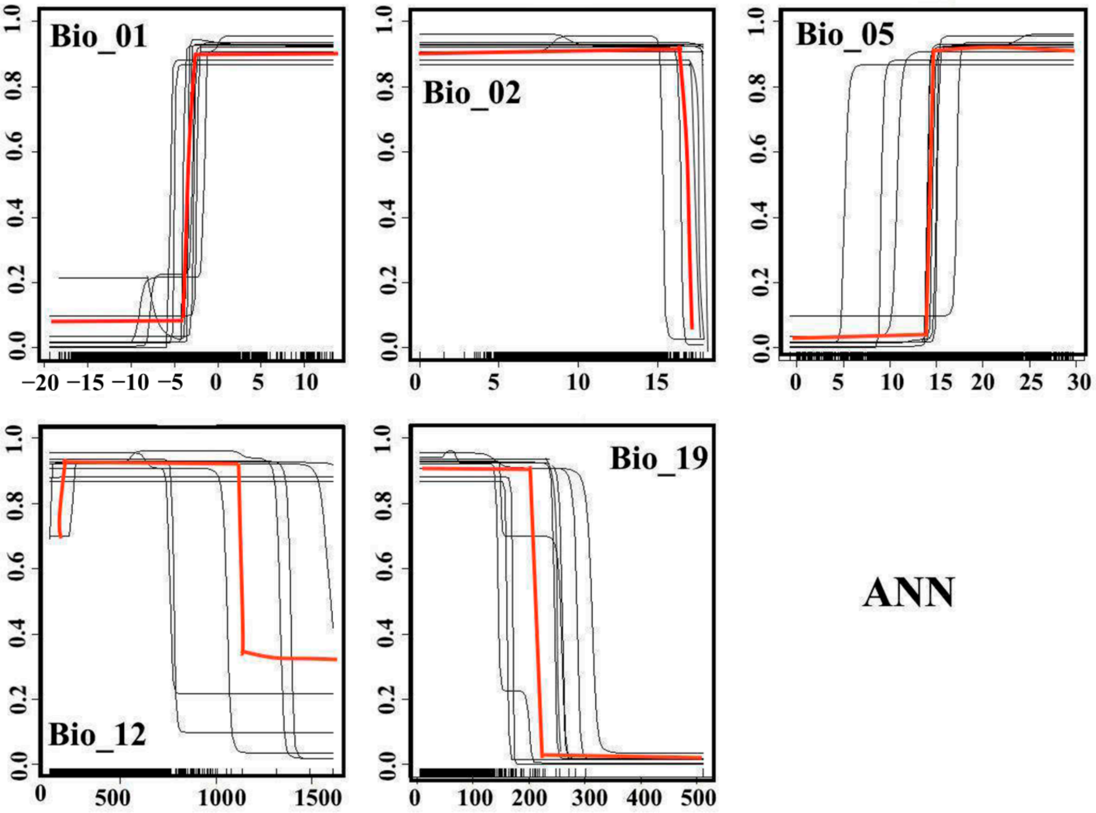

Figure S19. Plot of the response curves of five variables in ANN models for *Apodemus agrarius*.

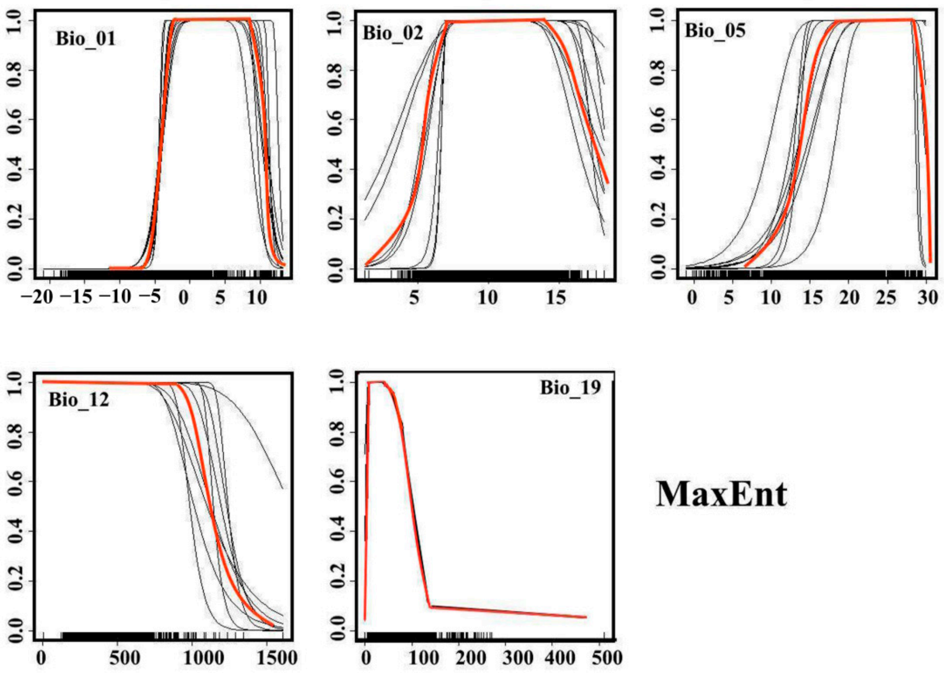

Figure S20. Plot of the response curves of five variables in MaxEnt models for *Apodemus agrarius*.

Table S1. Spearman's correlation matrix between predictor variables and VIF (Variation Inflation Factor) for every predictor variables.

Variables	Bio_01	Bio_02	Bio_05	Bio_12	Bio_19	VIF
Bio_01	1.00	0.07	0.7	0.72	0.6	4.25
Bio_02		1.00	0.52	0.06	−0.4	2.81
Bio_05			1.00	0.40	0.12	4.1
Bio_12				1.00	0.68	3.51
Bio_19					1.00	3.59

Table S2. Assessment of predicted mean annual temperature (°C) for three groups of GCMs (Hsens, Msens, Lsens) under four climate change scenarios (SSP1-2.6, SSP2-4.5, SSP3-7.0, SSP5-8.5) in 2021-2100.

GCMs	Scenarios	Years				
		1970–2000	2021–2040	2041–2060	2061–2080	2081–2100
Hsens*	SSP1-2.6	−4.82	−3.20	−2.45	−2.12	−1.90
	SSP2-4.5		−3.23	−1.94	−0.76	0.13
	SSP3-7.0		−3.28	−1.55	0.39	2.51
	SSP5-8.5		−2.94	−1.01	1.47	4.41
	Mean		−3.16(±0.15)	−1.74(±0.61)	−0.26(±1.54	1.29(±2.75)
	Change(±SD)		1.66(±0.15)	3.08(±0.61)	4.56(±1.54)	6.11(±2.75)
Msens**	SSP1-2.6	−4.82	−3.37	−2.84	−2.45	−2.64
	SSP2-4.5		−3.35	−2.30	−1.52	−1.03
	SSP3-7.0		−3.40	−2.13	−0.55	1.06
	SSP5-8.5		−3.04	−1.52	0.35	2.36
	Mean		−3.29(±0.17)	−2.20(±0.54)	−1.04(±1.21)	−0.06(±2.21)
	Change(±SD)		1.53(±0.17)	2.62(±0.54)	3.78(±1.21)	4.76(±2.21)
Lsens***	SSP1-2.6	−4.82	−3.69	−2.93	−2.67	−2.89
	SSP2-4.5		−3.46	−2.51	−1.93	−1.66
	SSP3-7.0		−3.61	−2.38	−1.12	0.08
	SSP5-8.5		−3.31	−1.90	−0.25	1.31
	Mean		−3.52(±0.17)	−2.43(±0.42)	−1.49(±1.14)	−1.49(±1.04)
	Change(±SD)		1.30(±0.17)	2.39(±0.42)	3.27(±1.14)	3.33(±1.04)

*Hsens includes: CanESM5, CNRM-CM6-1, CNRM-ESM2-1. IPSL-CM6A-LR GCMs; **Msens includes: CNRM-CM6-1-HR, EC-Earth3-Veg, MRI ESM2-0, BCC-CSM2-MR GCMs; Lsens*** includes: MIROC-ES2L, MIROC6, GISS-E2-1-G, INM-CM4.8 GCMs.

Table S3. Assessment of predicted means of variables for three groups of GCMs (Hsens, Msens, Lsens) under climate change scenarios SSPx-y (SSP1-2.6, SSP2-4.5, SSP3-7.0, SSP5-8.5) in 2021-2100.

GCMs	Predictor variables	Parameters	Years				
			1970–2000	2021–2040	2041–2060	2061–2080	2081–2100
All	Bio_01	Mean	−4.82	−3.32 (±0.15)	−2.12(±0.52)	−0.93(±1.22)	0.14(±2.27)
		Change		1.5(±0.15)	2.70(±0.52)	3.89(±1.22)	4.97(±2.27)
	Bio_02	Mean	9.73	9.71(±0.01)	9.55(±0.08)	9.41(±0.17)	9.30(±0.3)
		Change		−0.02(±0.01)	−0.17(±0.08)	−0.31(±0.17)	−0.42(±0.3)
	Bio_05	Mean	18.7	22.2(±0.1)	23.2(±0.4)	24.1(±1.0)	25.1(±1.9)
		Change		3.5(±0.1)	4.4(±0.4)	5.4(±1.0)	6.4(±1.9)
	Bio_12	Mean	430	458(±2)	468(±3)	478(±9)	487(±16)
		Change		27(±2)	38(±3)	48(±9)	56(±16)
	Bio_19	Mean	63.1	66.7(±0.1)	68.4(±0.8)	70.5(±2.0)	72.8(±4.2)
		Change		3.6(±0.1)	5.5(±0.8)	7.4(±2.0)	9.6(±4.2)
	De Martonne Index*		8.30	6.86	5.94	5.27	4.80
Hsens	Bio_01	Mean	−4.82	−3.16(±0.15)	−1.74(±0.61)	−0.26(±1.54	1.29(±2.75)
		Change		1.66(±0.15)	3.08(±0.61)	4.56(±1.54)	6.11(±2.75)
	Bio_02	Mean	9.73	9.54(±0.02)	9.30(±0.09)	9.08(±0.21)	8.86(±0.34)
		Change		−0.23(±0.02)	−0.54(±0.09)	−0.88(±0.21)	−1.26(±0.34)
	Bio_05	Mean	18.7	22.1(±0.1)	23.2(±0.5)	24.4(±1.2)	25.6(±2.3)
		Change		3.4(±0.1)	4.5(±0.5)	5.6(±1.2)	6.9(±2.3)
	Bio_12	Mean	430	455(±2)	466(±4)	478(±11)	492(±19)
		Change		25(±2)	36(±4)	48(±11)	61(±19)
	Bio_19	Mean	63.1	67.0(±0.3)	69.3(±1.1)	71.8(±2.8)	75.5(±6.1)
		Change		3.8(±0.3)	6.2(±1.1)	8.7(±2.8)	12.3(±6.1)
	De Martonne Index		8.30	6.65	5.64	4.91	4.36
Msens	Bio_01	Mean	−4.82	−3.29(±0.17)	−2.20(±0.54)	−1.04(±1.21)	−0.06(±2.21)
		Change		1.53(±0.17)	2.62(±0.54)	3.78(±1.21)	4.76(±2.21)
	Bio_02	Mean	9.73	9.73(±0.03)	9.58(±0.08)	9.46(±0.16)	9.38(±0.29)
		Change		0.00(±0.03)	−0.15(±0.08)	−0.27(±0.16)	−0.35(±0.29)
	Bio_05	Mean	18.7	22.3(±0.02)	23.2(±0.47)	24.1(±1.04)	25.1(±1.81)
		Change		3.6(±0.02)	4.4(±0.47)	5.4(±1.04)	6.4(±1.81)
	Bio_12	Mean	430	463(±3)	474(±4)	484(±10)	492(±18)
		Change		32(±3)	44(±4)	53(±10)	62(±18)
	Bio_19	Mean	63.1	66.8(±0.3)	68.4(±0.7)	70.2(±1.9)	72.1(±3.6)
		Change		3.7(±0.3)	5.3(±0.7)	7.0(±1.9)	9.0(±3.6)
	De Martonne Index(IDM)		8.30	6.90	6.08	5.40	4.95
Lsens	Bio_01	Mean	−4.82	−3.52(±0.17)	−2.43(±0.42)	−1.49(±1.04)	−0.79(±1.86)
		Change		1.30(±0.17)	2.39(±0.42)	3.33(±1.04)	4.03(±1.86)
	Bio_02	Mean	9.73	9.87(±0.02)	9.76(±0.07)	9.68(±0.14)	9.64(±0.21)
		Change		0.13(±0.02)	0.03(±0.07)	−0.06(±0.14)	−0.09(±0.21)
	Bio_05	Mean	18.7	22.3(±0.1)	23.2(±0.3)	23.9(±0.9)	24.7(±1.6)
		Change		3.6(±0.1)	4.4(±0.3)	5.2(±0.9)	5.9(±1.6)
	Bio_12	Mean	430	455(±1)	464(±3)	472(±7)	477(±12)
		Change		25(±1)	34(±3)	41(±7)	47(±12)
	Bio_19	Mean	63.1	66.3(±0.24)	68.2(±0.7)	69.1(±1.5)	70.7(±3.1)
		Change		3.3(±0.24)	5.0(±0.7)	6.4(±1.5)	7.6(±3.1)
	De Martonne Index		8.30	7.02	6.13	5.55	5.18

*De Martonne Index =P/(Ta+10), where P is annual precipitation (in millimeters)(Bio_12) and Ta is annual mean temperature (in degrees Celsius)(Bio_01)

Table S4. Results of a three-factor GLM ANOVA analysis of range changes in *A. agrarius* using gain, loss, and change metrics under GCMs and scenarios.

GLM ANOVA statistics	Source	Sum of Squares	Df	Mean Square	F-Ratio	P-Value
Gain, % (F=167.6 P<<0.001; R^2= 99.6 %)	Models	3254	2	1627	151.8	<<0.01
	Scenarios	1241	3	4136	384.5	<<0.01
	Years	2816	3	9387	872.7	<<0.01
	Models×Scenarios	214.5	6	35.8	3.32	0.02
	Models×Years	942.5	6	157.1	14.60	<<0.01
	Scenarios×Years	7314	9	812.7	75.55	<<0.01
	Residual	193.6	18	10.8		
	Total (corrected)	5249	47			
Loss, % (F=2.43, P=0.03; R^2=79.5 %)	Models	5.532	2	2.77	3.83	0.04
	Scenarios	16.8	3	5.60	7.75	0.002
	Years	13.31	3	4.44	6.14	0.005
	Models×Scenarios	1.877	6	0.31	0.43	0.85
	Models×Years	2.977	6	0.50	0.69	0.66
	Scenarios×Years	10.37	9	1.15	1.60	0.19
	Residual	13.0	18	0.72		
	Total (corrected)	63.86	47			
Change, % (F=171.2, P<<0.01, R^2=99.6%)	Models	3082	2	1541	140.2	<<0.01
	Scenarios	1329	3	4430	403.2	<<0.01
	Years	2928	3	9761	888.3	<<0.01
	Models×Scenarios	243.6	6	40.6	3.70	0.01
	Models×Years	946.0	6	157.7	14.4	<<0.01
	Scenarios×Years	7718	9	857.5	78.0	<<0.01
	Residual	197.8	18	10.99		
	Total (corrected)	5476	47			

References

1. Warren, D.L.; Glor, R.E.; Turelli, M. ENMTools: a toolbox for comparative studies of environmental niche models. *Ecography* **2010**, 33, 607-611.
2. Guisan, A.; Thuiller, W.; Zimmermann, N.E. *Habitat suitability and distribution models*. Cambridge University Press: Cambridge, United Kingdom, 2017.
3. Muscarella, R.; Galante, P.J.; Soley-Guardia, M.; Boria, R.A.; Kass, J.M.; Uriarte, M.; Anderson, R.P. ENMeval: An R package for conducting spatially independent evaluations and estimating optimal model complexity for Maxent ecological niche models*. Methods Ecol. Evol.* **2014**, 5, 1198–1205.
4. Halvorsen, R.; Mazzoni, S.; Dirksen, J.; Nasset, E.; Gobakken, T.; Ohlson, M. How important are choice of model selection method and spatial autocorrelation of presence data for distribution modelling by MaxEnt? *Ecol. Modell.* **2016**, 328: 108–118.
5. Petrosyan, V.; Osipov, F.; Feniova, I.; Dergunova, N.; Warshavsky, A.; Khlyap, L.; Dzialowski, A. The TOP-100 most dangerous invasive alien species in Northern Eurasia: invasion trends and species distribution modelling. *NeoBiota* **2023**, 82, 23-56.
6. Petrosyan, V.G.; Osipov, F.A.; Bobrov, V.V.; Dergunova, N.N.; Danielyan, F.D.; Arakelyan, M.S. Analysis of geographical distribution of the parthenogenetic rock lizard Darevskia armeniaca and its parental species (D. mixta, D. valentini) based on ecological modelling. *Salamandra* **2019**, 55, 173–190.
7. Petrosyan, V.; Osipov, F; Bobrov, V.; Dergunova, N.; Omelchenko, A.; Varshavskiy, A.; Danielyan, F.; Arakelyan, M. Species Distribution models and niche partitioning among unisexual Darevskia dahli and its parental bisexual (D. portschinskii, D. mixta) rock lizards in the Caucasus. *Mathematics* **2020**, 8, 1325.
